# The incidence of stroke among selected patients undergoing elective posterior lumbar fusion: a retrospective cohort study

**DOI:** 10.1186/s12891-020-03631-5

**Published:** 2020-09-14

**Authors:** Patrick J. Arena, Jingping Mo, Charu Sabharwal, Elizabeth Begier, Xiaofeng Zhou, Alejandra Gurtman, Qing Liu, Rongjun Shen, Charles Wentworth, Kui Huang

**Affiliations:** 1 Global Medical Epidemiology & Big Data Analysis, Pfizer Inc, New York, NY USA; 2 Safety Surveillance Research, Pfizer Inc, New York, NY USA; 3 Vaccine Research & Development, Pfizer Inc, Pearl River, NY USA; 4 Global Medical Epidemiology & Big Data Analysis, Pfizer Inc, Collegeville, PA USA

**Keywords:** Stroke, Posterior lumbar fusion, Epidemiology, Electronic healthcare records

## Abstract

**Background:**

Although stroke is a rare complication among spinal surgery patients, the recognition of this adverse event is critical given the aging population undergoing surgical procedures. The objective of this study was to estimate the incidence of stroke among selected adults undergoing elective posterior lumbar fusion (PLF) during various post-operative risk windows and among different subgroups.

**Methods:**

A retrospective cohort study using a longitudinal electronic healthcare record (EHR) database was conducted from January 1, 2007 to June 30, 2018. Elective PLF, stroke, and select clinical characteristics were defined based on International Classification of Disease codes. Patients aged 18 to 85 years with ≥183 days of enrollment in the database prior to undergoing elective PLF were followed from the index date until the occurrence of stroke, death, loss to follow-up, or end of study period, whichever occurred first. The incidence of stroke was estimated in the following risk windows: index hospitalization, ≤ 30 days, ≤ 90 days, ≤ 180 days, and ≤ 365 days post-operation.

**Results:**

A total of 43,063 patients were eligible for the study. The incidence of stroke following elective PLF was 0.29% (95% confidence interval [CI]: 0.25, 0.35%) during index hospitalization, 0.44% (95% CI: 0.38, 0.50%) ≤ 30 days, 0.59% (95% CI: 0.52, 0.67%) ≤ 90 days, 0.76% (95% CI: 0.68, 0.85%) ≤ 180 days, and 1.12% (95% CI: 1.03, 1.23%) ≤ 365 days post-operation. Stratified analyses revealed that older patients had a higher incidence of stroke. Additionally, black patients had higher stroke incidences. Post-operative stroke incidence was higher among patients with a history of type 2 diabetes than among patients without such history; similarly, stroke incidence was higher among patients with a history of stroke compared to patients without such history.

**Conclusions:**

The incidence of stroke following elective PLF using an EHR database in this study is slightly higher than that reported in the literature. Our results suggest that stroke risk modification prior to PLF may be important for patients who are older, black, type 2 diabetic, and/or have a history of stroke.

## Introduction

Stroke was the second leading cause of death globally in 2016, with 177,196 stroke deaths and 731,256 incident cases in the United States (US) alone [1]. Stroke is associated with increased economic burden due to costs associated with treatment and post-stroke care as evidenced by a recent review by Rasjic et al. in which overall post-stroke care costs were estimated to be $4850 per patient month in the US [2]. A 2017 review by Yang et al. concluded that hemorrhagic stroke after spine and joint surgeries is relatively rare, but it may cause serious consequences such as morbidity and mortality in the post-operative setting [3].

Although stroke is a rare complication among spinal surgery patients, the recognition of this adverse event is critical given the aging population undergoing surgical procedures. The volume of elective lumbar fusion procedures in the US has increased 62.3%, from 122,679 cases in 2004 to 199,140 cases in 2015; these increases were greatest among those aged 65 years or older [4]. Furthermore, Etzioni et al. have demonstrated that the number of older people (i.e., those above the age of 65 years) undergoing surgery in general is increasing at a rate faster than the proportion of older people in the overall population [5]. The estimated incidence of post-operative stroke in the spinal surgery setting varies widely depending on the type of surgical procedure and patient population. For adults undergoing posterior lumbar fusions (PLFs), the incidence of stroke within 30 days post-surgery has been reported as approximately 0.2% [6–9], but there is little data during various post-operative risk windows.

It is important to have background epidemiology data about post-operative stroke during various risk windows and among different subgroups to contextualize safety data in clinical trials and to better understand stroke in the general population. This cohort study was thus designed to estimate the incidence of stroke among selected adults undergoing elective PLF using a large electronic healthcare record (EHR) database in the US. The objectives of this study were to 1) estimate the incidence of stroke among elective PLF patients in various post-operative time periods (including the index surgical hospitalization) and 2) characterize the cohort’s demographic and clinical characteristics observed during the baseline to potentially identify those at increased risk of stroke.

## Methods

### Study design

A retrospective cohort study of adults undergoing elective PLF using a longitudinal EHR database, Optum EHR, was performed. Optum EHR partners directly with several multi-specialty medical groups, integrated delivery networks, and hospital chains throughout the US to extract their EHR data. By normalizing, validating, and aggregating the de-identified data, the database generates a longitudinal view of patient care and captures a comprehensive collection of demographic, clinical, operational, and financial information. As of June 30, 2017, Optum EHR reported having data on approximately 81 million unique patients. Furthermore, about 40% of the patient population was aged 50 years or older, with approximately 15% of patients 65 years of age or older. Almost one quarter (24%) of the patients had at least 6 years of observation time within the database.

The study period was January 1, 2007 to June 30, 2018 with a length of follow-up equating to 365 days. The index surgical date was defined as the first date on or after January 1, 2007 that an adult had undergone an elective PLF. To incorporate a 183-day look-back window prior to the index surgery (for the purpose of excluding prevalent conditions), the earliest possible index surgical date was July 1, 2007. The latest index surgical date possible was June 30, 2017.

### Cohort formation

Eligible patients were 18 to 85 years of age at the time of their first elective PLF. Furthermore, patients had 183 days of continuous enrollment within the database prior to their first elective PLF (i.e., the baseline period) as well as this index surgery being performed on the day of admission to the healthcare facility or the day after admission. PLF was identified using the following six International Classification of Diseases (ICD), Ninth Revision, Procedure Classification System (ICD-9-PCS) codes: 81.05 (dorsal and dorsolumbar fusion of the posterior column, posterior technique), 81.07 (lumbar and lumbosacral fusion of the posterior column, posterior technique), 81.08 (lumbar and lumbosacral fusion of the anterior column, posterior technique), 81.35 (refusion of dorsal and dorsolumbar spine, posterior column, posterior technique), 81.37 (refusion of lumbar and lumbosacral spine, posterior column, posterior technique), and 81.38 (refusion of lumbar and lumbosacral spine, anterior column, posterior technique).

To select for a healthy cohort that underwent inpatient elective surgeries, patients were excluded if they 1) underwent a major surgical procedure that occurred within 90 days prior to the index surgery; 2) had a surgical indication that was for an emergency procedure; 3) were pregnant; 4) were discharged on the same date of the index surgery (thereby indicating an outpatient procedure); 5) had any of the following conditions during the baseline period: anaphylactic reaction to a vaccine, cancer, end stage renal disease, congenital spleen anomalies, an immunosuppressive state, and/or receipt of corticosteroids or immunosuppressive medications; or 6) had any of the following conditions at the time of the index surgery: potential/presumed surgical site-related infection and/or spinal infection.

Stroke was defined based on the following ten ICD, Ninth Revision, Clinical Modification (ICD-9-CM) codes: 433.01 (occlusion and stenosis of basilar artery with cerebral infarction), 433.11 (occlusion and stenosis of carotid artery with cerebral infarction), 433.21 (occlusion and stenosis of vertebral artery with cerebral infarction), 433.31 (occlusion and stenosis of multiple and bilateral precerebral arteries with cerebral infarction), 433.81 (occlusion and stenosis of other specified precerebral artery with cerebral infarction), 433.91 (occlusion and stenosis of unspecified precerebral artery with cerebral infarction), 434.01 (cerebral thrombosis with cerebral infarction), 434.11 (cerebral embolism with cerebral infarction), 434.91 (cerebral artery occlusion, unspecified with cerebral infarction), and 436 (acute, but ill-defined, cerebrovascular disease). ICD-9-PCS and ICD-9-CM codes were mapped to corresponding ICD, Tenth Revision (ICD-10) codes using General Equivalence Mapping techniques in order to account for the switch to ICD-10 coding in 2015.

### Data management and analysis

All analyses were descriptive and conducted in SAS (version 9.4, SAS Institute, Cary, NC, USA). Descriptive statistics were performed to characterize the cohort in terms of demographic and clinical characteristics at the baseline. Patients were followed from the cohort entry index date until the occurrence of stroke, death, loss to follow-up, or end of study period, whichever occurred first.

Incidence was defined as the number of new cases of stroke during each specified time interval divided by the total (stroke-free) population at the start of each time interval; thus, the incidences calculated here are incidence proportions. Crude incidence was calculated overall, and in the following stratifications: age, sex, race, length of hospital stay, and selected clinical characteristics. Incidence was also estimated in the following risk windows: index hospitalization (defined as the time interval from index surgery to discharge), ≤ 30 days (i.e., 0 to 30 days), ≤ 90 days (i.e., 0 to 90 days), ≤ 180 days (i.e., 0 to 180 days), and ≤ 365 days (i.e., 0 to 365 days) post-operation. For each post-operation period, incidence was calculated cumulatively; therefore, persons at risk and stroke events that were included in the preceding risk window were not excluded in the incidence calculation for the following risk window. Incidences were estimated with associated 95% confidence intervals (CIs), assuming a Poisson distribution.

Incidence rates were also produced and were calculated as the number of new cases of stroke during each specified time interval divided by the summed person-time of observation for the total (stroke-free) population at the start of each time interval. Although incidence rates are preferred over incidence proportions when there is long-term follow up (i.e., > 30 days), nearly all the literature identified in this area presented information in the form of incidence proportions; thus, only incidence proportions are presented in the Results in order to facilitate better comparisons with the literature. However, incidence rate information is contained in Additional file 1.

## Results

Of the 80,796 patients who were 18 to 85 years of age with at least one record of elective PLF during the study period and adequate prior enrollment in the database, 37,733 met exclusion criteria; the most common reasons for exclusion were use of immunosuppressive medications (42%), receipt of systemic corticosteroids (33%), and a diagnosis of cancer (14%) during the baseline. Ultimately, 43,063 patients were included for analysis; 42,966 patients had at least 365 days of follow-up from the index date (thereby indicating a low level of loss-to-follow-up in the study). The mean age was 59.4 years, and there were slightly more females (52.22%) than males (47.75%). The majority of the cohort members were white (89.49%), while black (5.94%) and Asian (0.52%) members were less represented. The most prevalent medical conditions were type 2 diabetes (13.86%), cardiac dysrhythmias (9.28%), and chronic ischemic disease (9.00%). Only 293 patients (0.68%) had any history of stroke during the baseline. Dementia, individual digestive disorders, deep vein thrombosis, and pulmonary embolism were also rare (i.e., each less than 1.00%). Moreover, the average length of hospital stay (for the index hospitalization) was 3.8 days. Lastly, no patients died during initial hospitalization; by the end of the study period, all-cause mortality was approximately 0.50%. Table 1 shows the baseline demographics and clinical characteristics of the elective PLF patient population.

**Table 1:** Baseline demographics, clinical characteristics, and surgical characteristics of the elective posterior lumbar fusion patient population

Characteristic, N (%) except where specified	Number of patients	%
**Total**	43,063	
**Demographic characteristics**		
**Age (years) at index date**		
*Mean (SD)*	59.4 (13.69)	
*Median (Range)*	61 (18, 85)	
≥ 18–55	15,221	35.35
56–65	11,639	27.03
66–75	11,579	26.89
76 - < 86	4624	10.74
**Race**		
White	38,535	89.49
Black or African American	2556	5.94
Asian	223	0.52
Other/Unknown	1749	4.06
**Sex**		
Male	20,563	47.75
Female	22,487	52.22
Unknown	13	0.03
**Clinical & surgical characteristics in 183-day baseline**		
**Arthritis and other inflammation (or rheumatic events)**		
Rheumatoid arthritis	0	0.00
Reactive arthritis	579	1.34
Psoriatic arthroplasty	42	0.10
Spondyloarthritis, including ankylosing spondylitis	1476	3.43
**Blood disorders**		
Anemia/other anemia	3494	8.11
Intracranial Hemorrhage	52	0.12
Gastrointestinal bleeding	216	0.50
Thrombocytopenia	333	0.77
**Cardiovascular events and/or conditions**		
Acute myocardial infarction	270	0.63
Angina	1997	4.64
Cardiac dysrhythmias	3997	9.28
Stroke	293	0.68
Chronic ischemic disease	3876	9.00
Peripheral vascular disease	1811	4.21
**Dementia**		
Dementia	130	0.30
**Diabetes**		
Diabetes, type 1	272	0.63
Diabetes, type 2	5970	13.86
**Digestive disorders**		
Crohn’s disease	73	0.17
Ulcerative colitis	71	0.16
Peptic ulcer disease	240	0.56
**Hepatic disorders**		
Liver disease and cirrhosis	607	1.41
**Nervous system disorders**		
Guillain-Barre syndrome	0	0.00
Multiple sclerosis	105	0.24
**Respiratory disorders**		
Asthma/wheezing/bronchospasm	3353	7.79
Bronchitis/chronic obstructive pulmonary disease	2315	5.38
Obstructive asthma	1126	2.61
**Surgical characteristics**		
Existing permanently implanted device or prosthesis at baseline	1270	2.95
Total length of hospital stay		
*Mean (SD)*	3.8 (3.4)	
*Median (Min, Max)*	3 (1, 90)	
History of allogenic blood transfusion during surgery	1	0.00
Revisional surgery on the same day as index surgery	320	0.74
Use of implanted material during surgery on the same day as index surgery	2667	6.19
**Thrombotic events**		
Deep vein thrombosis	386	0.90
Pulmonary embolism	170	0.39
**Thyroid disorders**		
Grave’s disease	37	0.09
Autoimmune thyroiditis	55	0.13
Hyperthyroidism	109	0.25
Hypothyroidism	3455	8.02
Thyroiditis	6	0.01

The crude incidence of stroke following elective PLF was 0.29% (95% CI: 0.25, 0.35%) during index hospitalization, 0.44% (95% CI: 0.38, 0.50%) ≤ 30 days, 0.59% (95% CI: 0.52, 0.67%) ≤ 90 days, 0.76% (95% CI: 0.68, 0.85%) ≤ 180 days, and 1.12% (95% CI: 1.03, 1.23%) ≤ 365 days post-operation. Table 2 shows both crude and stratified stroke incidences in patients undergoing elective PLF.

**Table 2:** Incidence of stroke in patients undergoing elective posterior lumbar fusion during various risk windows

Stroke incidence type	During index hospitalization				Up to 30 days post-operation				Up to 90 days post-operation				Up to 180 days post-operation				Up to 365 days post-operation			
	Number of patients at risk	Number of cases	Years at risk	Incidence proportion(95% confidence interval)	Number of patients at risk	Number of cases	Years at risk	Incidence proportion(95% confidence interval)	Number of patients at risk	Number of cases	Years at risk	Incidence proportion(95% confidence interval)	Number of patients at risk	Number of cases	Years at risk	Incidence proportion(95% confidence interval)	Number of patients at risk	Number of cases	Years at risk	Incidence proportion(95% confidence interval)
**Crude**	42,742	126	550.03	0.29% (0.25, 0.35%)	42,744	186	3523.41	0.44% (0.38, 0.50%)	42,745	252	9833.32	0.59% (0.52, 0.67%)	42,746	327	18,712.01	0.76% (0.68, 0.85%)	42,750	480	35,079.03	1.12% (1.03, 1.23%)
*Stratifications by:*																				
**Age (years)**																				
≥ 18–55	15,181	17	179.62	0.11% (0.07, 0.18%)	15,181	25	1251.95	0.16% (0.11, 0.24%)	15,181	36	3498.67	0.24% (0.17, 0.33%)	15,181	44	6673.80	0.29% (0.21, 0.39%)	15,181	61	12,537.16	0.40% (0.31, 0.52%)
56–65	11,588	22	146.04	0.19% (0.12, 0.29%)	11,590	39	956.04	0.34% (0.24, 0.46%)	11,591	53	2662.86	0.46% (0.34, 0.60%)	11,591	70	5058.52	0.60% (0.47, 0.76%)	11,593	105	9446.15	0.91% (0.74, 1.10%)
66–75	11,438	51	157.62	0.45% (0.33, 0.59%)	11,438	71	942.84	0.62% (0.49, 0.78%)	11,438	90	2634.55	0.79% (0.63, 0.97%)	11,438	127	5009.82	1.11% (0.93, 1.32%)	11,440	190	9383.93	1.66% (1.43, 1.91%)
76 - < 86	4535	36	66.74	0.79% (0.56, 1.10%)	4535	51	372.57	1.12% (0.84, 1.48%)	4535	73	1037.24	1.61% (1.26, 2.02%)	4536	86	1969.88	1.90% (1.52, 2.34%)	4536	124	3711.79	2.73% (2.28, 3.25%)
**Sex**																				
Male	20,404	64	256.25	0.31% (0.24, 0.40%)	20,405	96	1680.10	0.47% (0.38, 0.57%)	20,405	129	4681.68	0.63% (0.53, 0.75%)	20,406	175	8898.68	0.86% (0.74, 0.99%)	20,409	258	16,640.24	1.26% (1.12, 1.43%)
Female	22,325	62	293.61	0.28% (0.21, 0.36%)	22,326	90	1842.21	0.40% (0.32, 0.50%)	22,327	123	5148.73	0.55% (0.46, 0.66%)	22,327	152	9807.71	0.68% (0.58, 0.80%)	22,328	222	18,427.89	0.99% (0.87, 1.13%)
Unknown	13	0	0.17	0.00% (0.00, 0.00%)	13	0	1.10	0.00% (0.00, 0.00%)	13	0	2.91	0.00% (0.00, 0.00%)	13	0	5.62	0.00% (0.00, 0.00%)	13	0	10.90	0.00% (0.00, 0.00%)
**Race**																				
White	38,246	113	487.13	0.30% (0.24, 0.36%)	38,248	160	3154.63	0.42% (0.36, 0.49%)	38,249	218	8814.21	0.57% (0.50, 0.65%)	38,250	286	16,787.98	0.75% (0.66, 0.84%)	38,254	419	31,522.14	1.10% (0.99, 1.20%)
Black or African American	2545	9	36.89	0.35% (0.16, 0.67%)	2545	17	210.81	0.67% (0.39, 1.07%)	2545	23	585.70	0.90% (0.57, 1.35%)	2545	28	1108.84	1.10% (0.73, 1.59%)	2545	42	2063.04	1.65% (1.19, 2.22%)
Asian	212	0	3.33	0.00% (0.00, 0.00%)	212	0	17.43	0.00% (0.00, 0.00%)	212	1	49.07	0.47% (0.01, 2.60%)	212	2	93.98	0.94% (0.11, 3.37%)	212	3	172.13	1.42% (0.29, 4.08%)
Other/Unknown	1739	4	22.68	0.23% (0.06, 0.59%)	1739	9	140.54	0.52% (0.24, 0.98%)	1739	10	384.34	0.58% (0.28, 1.06%)	1739	11	721.21	0.63% (0.32, 1.13%)	1739	16	1321.71	0.92% (0.53, 1.49%)
**Type 1 Diabetes during baseline**																				
Yes	270	1	3.85	0.37% (0.01, 2.05%)	270	2	22.56	0.74% (0.09, 2.65%)	271	3	64.50	1.11% (0.23, 3.20%)	271	4	123.85	1.48% (0.40, 3.74%)	271	9	231.14	3.32% (1.53, 6.21%)
No	42,472	125	546.17	0.29% (0.25, 0.35%)	42,474	184	3500.85	0.43% (0.37, 0.50%)	42,474	249	9768.82	0.59% (0.52, 0.66%)	42,475	323	18,588.16	0.76% (0.68, 0.85%)	42,479	471	34,847.88	1.11% (1.01, 1.21%)
**Type 2 Diabetes during baseline**																				
Yes	5923	23	82.24	0.39% (0.25, 0.58%)	5923	42	492.66	0.71% (0.51, 0.96%)	5924	58	1394.58	0.98% (0.74, 1.26%)	5924	81	2672.70	1.37% (1.09, 1.70%)	5926	123	5026.02	2.08% (1.73, 2.47%)
No	36,819	103	467.78	0.28% (0.23, 0.34%)	36,821	144	3030.75	0.39% (0.33, 0.46%)	36,821	194	8438.74	0.53% (0.46, 0.61%)	36,822	246	16,039.31	0.67% (0.59, 0.76%)	36,824	357	30,053.01	0.97% (0.87, 1.07%)
**Existing permanently implanted device or prosthesis at baseline**																				
Yes	1242	0	17.80	0.00% (0.00, 0.00%)	1242	1	102.71	0.08% (0.00, 0.45%)	1242	1	287.33	0.08% (0.00, 0.45%)	1242	4	547.00	0.32% (0.09, 0.82%)	1242	8	1019.47	0.64% (0.28, 1.27%)
No	41,500	126	532.23	0.30% (0.25, 0.36%)	41,502	185	3420.70	0.45% (0.38, 0.51%)	41,503	251	9545.98	0.60% (0.53, 0.68%)	41,504	323	18,165.01	0.78% (0.70, 0.87%)	41,508	472	34,059.56	1.14% (1.04, 1.24%)
**Total length of hospital stay**																				
1–5 days	37,077	60	393.57	0.16% (0.12, 0.21%)	37,079	104	3053.66	0.28% (0.23, 0.34%)	37,080	148	8514.74	0.40% (0.34, 0.47%)	37,081	209	16,198.14	0.56% (0.49, 0.65%)	37,084	336	30,364.03	0.91% (0.81, 1.01%)
6–10 days	4530	43	98.86	0.95% (0.69, 1.28%)	4530	57	375.63	1.26% (0.95, 1.63%)	4530	70	1054.52	1.55% (1.21, 1.95%)	4530	80	2012.51	1.77% (1.40, 2.19%)	4530	99	3774.44	2.19% (1.78, 2.65%)
> 10 days	1135	23	57.60	2.03% (1.29, 3.03%)	1135	25	94.11	2.20% (1.43, 3.23%)	1135	34	264.06	3.00% (2.08, 4.16%)	1135	38	501.35	3.35% (2.38, 4.57%)	1136	45	940.55	3.96% (2.90, 5.26%)
**Use of implanted material during surgery on the same day as index surgery**																				
Yes	3095	11	44.45	0.36% (0.18, 0.64%)	3095	16	255.45	0.52% (0.30, 0.84%)	3095	19	714.26	0.61% (0.37, 0.96%)	3095	32	1373.09	1.03% (0.71, 1.46%)	3095	44	2626.26	1.42% (1.03, 1.90%)
No	39,647	115	505.58	0.29% (0.24, 0.35%)	39,649	170	3267.96	0.43% (0.37, 0.50%)	39,650	233	9119.06	0.59% (0.51, 0.67%)	39,651	295	17,338.92	0.74% (0.66, 0.83%)	39,655	436	32,452.77	1.10% (1.00, 1.21%)
**Medical history of stroke**																				
Yes	291	39	4.59	13.40% (9.71, 17.86%)	292	58	20.47	19.86% (15.44, 24.91%)	292	77	54.44	26.37% (21.41, 31.82%)	292	84	100.04	28.77% (23.64, 34.33%)	292	106	177.45	36.30% (30.78, 42.11%)
No	42,451	87	545.43	0.20% (0.16, 0.25%)	42,452	128	3502.94	0.30% (0.25, 0.36%)	42,453	175	9778.87	0.41% (0.35, 0.48%)	42,454	243	18,611.98	0.57% (0.50, 0.65%)	42,458	374	34,901.58	0.88% (0.79, 0.97%)

Stratified analyses revealed that older patients consistently had higher incidences of post-operative stroke during all surgical risk windows. For example, the incidence of post-operative stroke ≤365 days post-operation was 0.40% (95% CI: 0.31, 0.52%) among those aged ≥18 to 55 years, 0.91% (95% CI: 0.74, 1.10%) among those aged 56 to 65 years, 1.66% (95% CI: 1.43, 1.91%) among those aged 66 to 75 years, and 2.73% (95% CI: 2.28, 3.25%) among those aged 76 to < 86 years. Moreover, men had higher incidences of stroke than women; for instance, the incidence of post-operative stroke during index hospitalization was 0.31% (95% CI: 0.24, 0.40%) for men and 0.28% (95% CI: 0.21, 0.36%) for women. Additionally, black patients had higher stroke incidences than white, Asian, or other patients: the incidence of post-operative stroke ≤365 days post-operation was 1.10% (95% CI: 0.99, 1.20%) among white adults, 1.65% (95% CI: 1.19, 2.22%) among black adults, 1.42% (95% CI: 0.29, 4.08%) among Asian adults, and 0.92% (95% CI: 0.53, 1.49%) among other adults.

**Fig. 1:**
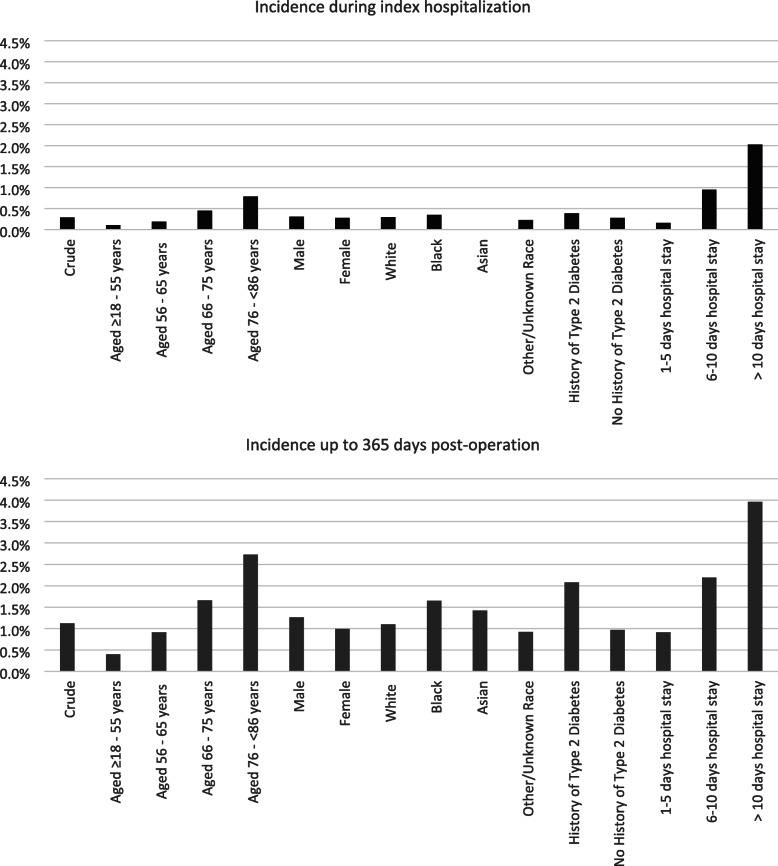
Incidence during index hospitalization and up to 365 days post-operation, by key demographic and clinical characteristics

The incidence of post-operative stroke was higher among patients with a history of type 2 diabetes than among patients without such history; for instance, the incidence of stroke was 2.08% (95% CI: 1.73, 2.47%) for those with a history of type 2 diabetes and 0.97% (95% CI: 0.87, 1.07%) for those without such history ≤365 days post-operation. The incidence of post-operative stroke was much higher among patients with a history of stroke than among patients without a history of stroke during all risk windows. For example, post-operative stroke incidence during index hospitalization was 13.40% (95% CI: 9.71, 17.86%) among those with a history of stroke and 0.20% (95% CI: 0.16, 0.25%) among those without such history. Lastly, patients with longer hospital stays had a higher incidence of stroke. However, it should be emphasized that these longer hospital stays could be a result of stroke instead of a reverse relationship (i.e., where stroke is the consequence of a longer hospital stay). Figure 1 displays relevant incidence results during index hospitalization and ≤ 365 days post-operation stratified by certain demographic and clinical characteristics.

Detailed incidence rate information can be found in Additional file 1. The crude incidence rate of stroke following elective PLF decreased consistently from index hospitalization to ≤365 days post-operation; these incidence rates per 1000 person-years were 229.08 (95% CI: 192.38, 272.78) during index hospitalization, 52.79 (95% CI: 45.72, 60.95) ≤ 30 days, 25.63 (95% CI: 22.65, 28.99) ≤ 90 days, 17.48 (95% CI: 15.68, 19.48) ≤ 180 days, and 13.68 (95% CI: 12.51, 14.96) ≤ 365 days post-operation.

## Discussion

### Summary

This study identified 43,063 eligible patients who were relatively healthy and underwent inpatient elective PLF surgeries within the Optum EHR database. The incidence of stroke following elective PLF ranged from 0.29% (95% CI: 0.25, 0.35%) during index hospitalization to 1.12% (95% CI: 1.03, 1.23%) ≤ 365 days post-operation. When stratified by relevant demographic and clinical characteristics, we found that age, race, type 2 diabetes status, and stroke history were associated with stroke incidence; more specifically, PLF patients who were older, black, type 2 diabetic, or had a history of stroke had increased risk of post-operative stroke.

### Stroke incidence

Minhas et al. examined the incidence of peri-operative cerebrovascular accidents (CVAs) among patients undergoing elective orthopedic procedures from 2006 to 2012 within the National Surgical Quality Improvement Program (NSQIP) database and reported that the 30-day incidence of CVA was 0.35% for single-level/multilevel PLF (*n* = 2895) [10]. In our study, a 30-day incidence for post-operative stroke of 0.44% (95% CI: 0.38, 0.50%) was observed; however, it should be noted the authors defined their procedures using Current Procedure Terminology (CPT) codes and their definition for CVA was based on medical record review [10]. In a 2012 retrospective database study, 2015 PLF patients were identified from a nationwide Taiwanese cohort from 2000 to 2005 using The National Health Insurance Research Database and followed up for 3 years. The incidence rate of stroke per 1000 person-years was 10.22 (95% CI: 7.94, 13.17). In their study, Wu et al. used ICD-9-CM codes 430–435 for stroke and ICD-9-PCS codes 81.0 and 81.38 to identify PLF [11]. The incidence rate of stroke per 1000 person-years in our study was 13.68 (95% CI: 12.51,14.96) ≤ 365 days post-operation, which was in line with their finding.

A 2014 retrospective cohort study performed by Marquez-Lara et al. used the Nationwide Inpatient Sample (NIS) database from 2002 to 2011 to identify patients undergoing elective lumbar fusion procedures and found a post-operative CVA incidence of 0.15% among the PLF patients (*n* = 214,837) [11]. In our study, the incidence of stroke following elective PLF ranged from 0.29% (95% CI: 0.25, 0.35%) to 1.12% (95% CI: 1.03, 1.23%). It should be noted that only patients with ICD-9 code 81.08 were included and that CVA was defined with only one ICD-9 code (997.02) in their study. However, the researchers also found that increased length of hospital stay was associated with post-operative CVA [12]. Furthermore, a variety of studies examined 30-day outcomes following lumbar spinal fusion within both the NIS and NSQIP databases and generally found that the 30-day overall incidence of post-operative stroke was about 0.20% [6–9, 13].

Our finding that the risk of stroke increased with age was consistent with the literature within the general population [14]. Furthermore, studies evaluating the risk of stroke among adults undergoing lumbar spinal fusion also found an association between increased age and the risk of stroke [10, 12, 15]. With regards to sex, Minhas et al. and Marquez-Lara et al. found no statistically significant difference in the sex-specific incidence of stroke [10, 12], which is in line with our findings. Stratified analyses revealed that black patients had a higher stroke incidence compared to other ethnic groups. This finding is consistent with the literature as numerous studies have illustrated that black patients have significantly higher risk of stroke in the general population [16–18]. Among populations undergoing elective PLF though, there was no published data on the incidence of post-operative stroke by race.

With regards to the other subgroups, limited information among PLF patients is available in the literature. However, Minhas et al. reported that patients with insulin-dependent diabetes mellitus (IDDM) had 3.08 times the odds of a post-operative CVA compared to those without IDDM [10]. Furthermore, recent reviews have demonstrated that diabetes mellitus is an established risk factor for stroke in the general population [19, 20]. Thus, our finding that post-operative stroke incidence was higher among patients with a history of type 2 diabetes is consistent with the literature. Lastly, it is known that the incidence of stroke among those with a medical history of stroke is increased (i.e., there is a high risk of recurrent stroke) [19, 20]; our results are thus in line with the standard medical knowledge regarding recurrent stroke.

### Implications and relevance

As peri-operative stroke complications are associated with longer hospitalizations and increased hospital costs in addition to long-term complications such as epilepsy, depression, and pain [4, 12, 21–26], these results suggest that appropriate stroke risk management prior to PLF may be needed for patients who are older, black, type 2 diabetic, and/or have a history of stroke. Lad et al. reached similar conclusions and stated that African American patients were more likely to experience postoperative complications of any kind for lumbar stenosis, even after adjusting for length of hospital stay, comorbidities, sex, and age [27]. Despite controversies surrounding appropriate peri-operative management of complicated medication regimens among elderly patients, healthcare providers should ensure that modifiable stroke risks are controlled and should include any concerns in discussions with their patients [28]. With regard to undiagnosed diabetes, a 2017 review by Epstein recommended routine pre-operative screening for diabetes with HbA1c levels among spinal surgery patients to facilitate pre-operative, intra-operative, and post-operative management [29]. However, the feasibility of such an approach would require that an appropriate referral mechanism already be in place.

Although anticoagulants might be a suitable prophylaxis to prevent stroke in a general surgical setting, it should be highlighted that patients undergoing spinal surgery while under anticoagulation therapy are at risk of developing bleeding complications [30]. A 2020 review of anticoagulation and spine therapy by Porto et al. stated that current practice suggests holding warfarin until international normalized ratio < 1.4, anti-Xa drugs for 48 to 72 h, 12 to 24 h for low-molecular-weight heparin, and 4 to 24 h for heparin, before surgery. For antiplatelet agents, current practice indicated that they can be stopped for 1 to 3 days prior to operation (81–500 mg) but must be stopped for 1 week for doses > 1 g/d. Current guidelines also recommended Plavix be discontinued for 5 to 7 days to prevent complications. Nonetheless, randomized control trials are needed in order to provide definitive guidance [31].

Moreover, this study provides additional information about stroke in a variety of risk windows. Most studies identified in the literature analyzed stroke events during index hospitalization or in the 30- or 90-day risk windows; our study thus builds on previous work by not only estimating stroke incidences during index hospitalization and the 30- and 90-day risk windows but also by generating data on stroke incidence in the 180- and 365-day risk windows. Lastly, our study adds to the existing literature about stroke incidence by presenting such information in the form of incidence rates (see Additional file 1); most of the stroke incidence information in the literature is presented in the form of incidence proportions, and thus there is a paucity of data in the form of incidence rates.

### Variable and database considerations

A 2012 review by Andrade et al. examined the validity of algorithms for identifying CVAs using administrative/claims data among 35 identified studies and ultimately concluded that the algorithms and definitions used to identify CVAs using administrative/claims data differ greatly in the published literature. However, the authors determined that studies reported the highest positive predictive values for inpatient ICD-9 codes 430.x, 431.x, 434.x, and 436.x for acute stroke while algorithms that included ICD-9 codes 433.× 1, 434 (excluding 434.× 0), and 436 performed well (85% or higher) for transient ischemic stroke [32].

Lastly, it is noteworthy that our study used Optum EHR while most studies in the literature used NSQIP or NIS. Because of differences in these data sources, it may not be surprising that our results would not exactly align with the incidence information found in the literature. For instance, a 2016 study evaluated the variability in standard outcomes of PLF between the University HealthSystem Consortium (UHC) and the NIS and found that the databases had similar patient populations undergoing PLF, but that the UHC database reported significantly higher complication rates and longer lengths of hospital [33]. Additionally, recent studies have also shown that certain variables have changed over time within both NIS and NSQIP [9, 15]; thus, even comparisons within the same database can be fraught.

### Strengths and limitations

The Optum EHR database has both inpatient and outpatient data as well as a large sample size that enabled us to generate real-world incidence estimates that are generalizable to a segment of the commercially insured US population (i.e., those in the Optum network). However, our patient population was selected to be relatively healthy, so this selection may affect the overall generalizability. Nonetheless, this study is one of the first to examine adverse outcomes among spinal surgery patients using an EHR database (as most studies in this area have used claims databases).

Still, it must be noted that EHR data were originally developed to improve patient care/modernize billing procedures and thus were not designed as research resources. As a result, EHR data tend to have more missing data (when compared to data obtained from clinical trials and/or prospective studies with primary data collection), and this missingness can potentially bias results [34]. However, given that elective surgery and stroke events generally require medical encounters, they would have been recorded in Optum EHR; therefore, the likelihood of missing information for these key variables would be very low. Like other studies utilizing secondary data sources without validation (e.g., medical chart review), exposure and outcome misclassification are also possible; diagnosis codes may have been incorrect or included as part of the diagnostic rule-out process rather than an indication of disease or surgery itself. Furthermore, patients may have sought healthcare outside Optum EHR prior to the index surgery, so it is possible that a patient developed a stroke prior to the index surgery; similarly, some incident events may have been missed if a patient sought care outside the system after surgery. Likewise, conditions identified during the baseline that do not require treatment or office visits (such as wheezing) tend to be systematically under-recorded in EHR databases; therefore, it is possible that this study only captured severe manifestations of such disorders. Additionally, as we used a broad set of ICD codes to identify stroke events, we may have overestimated its incidence.

Lastly, this study employed a descriptive analysis approach; thus, comparisons within stratified analyses may be subject to confounding factors that were not properly controlled. As a result, these comparisons must be interpreted with caution. Future studies in this area should consider multiple regression modeling and/or multivariable stratification techniques to better account for potential confounding.

## Conclusion

This study estimated the incidence of stroke using an EHR database among adults undergoing elective PLF during various post-operative risk windows and among different subgroups. This incidence is slightly higher than that reported in the literature; however, the discrepancy is due to differences in the variable definitions, study populations, follow-up periods, and data sources between our study and those in the literature. Our results suggest that appropriate stroke risk modification prior to PLF may be of particular importance for patients who are older, black, type 2 diabetic, and/or have a history of stroke.

## Supplementary information

**Additional file 1.** Incidence rate of stroke in patients undergoing elective posterior lumbar fusion during various risk windows

## Data Availability

The data that support the findings of this study are available from Optum but restrictions apply to the availability of these data, which were used under license for the current study, and so are not publicly available. Data are however available from the authors upon reasonable request and with permission of Optum.

## Abbreviations

CI: Confidence interval
CPT: Current Procedure Terminology
CVA: Cerebrovascular accident
EHR: Electronic healthcare record
ICD: International Classification of Diseases
ICD-9-PCS: International Classification of Diseases, Ninth Revision, Procedure Classification System
ICD-9-CM: International Classification of Diseases, Ninth Revision, Clinical Modification
ICD-10: International Classification of Diseases, Tenth Revision
IDDM: Insulin-dependent diabetes mellitus
NIS: Nationwide Inpatient Sample
NSQIP: National Surgical Quality Improvement Program
PLF: Posterior lumbar fusion
UHC: University HealthSystem Consortium
US: United States
