# Supplementary material for: The incidence of stroke among selected patients undergoing elective posterior lumbar fusion: a retrospective cohort study
Source: BMC Musculoskelet Disord. 2020 Sep 14;21:612. doi: 10.1186/s12891-020-03631-5 (PMC7488711; doi:10.1186/s12891-020-03631-5)
Supplement: Supplementary file 1 — Additional file 1. Incidence rate of stroke in patients undergoing elective posterior lumbar fusion during various risk windows [file 12891_2020_3631_MOESM1_ESM.docx]

Appendix

Incidence rate of stroke in patients undergoing elective posterior lumbar fusion during various risk windows

| **Stroke incidence type** | **During index hospitalization** | | | | **Up to 30 days post-operation** | | | | **Up to 90 days post-operation** | | | | **Up to 180 days post-operation** | | | | **Up to 365 days post-operation** | | | |
| --- | --- | --- | --- | --- | --- | --- | --- | --- | --- | --- | --- | --- | --- | --- | --- | --- | --- | --- | --- | --- |
|  | **Number of patients at risk** | **Number of cases** | **Years at risk** | **Incidence rate per 1000 person-years (95% confidence interval)** | **Number of patients at risk** | **Number of cases** | **Years at risk** | **Incidence rate per 1000 person-years (95% confidence interval)** | **Number of patients at risk** | **Number of cases** | **Years at risk** | **Incidence rate per 1000 person-years (95% confidence interval)** | **Number of patients at risk** | **Number of cases** | **Years at risk** | **Incidence rate per 1000 person-years (95% confidence interval)** | **Number of patients at risk** | **Number of cases** | **Years at risk** | **Incidence rate per 1000 person- years (95% confidence interval)** |
| **Crude** | 42,742 | 126 | 550.03 | 229.08 (192.38, 272.78) | 42,744 | 186 | 3523.41 | 52.79 (45.72, 60.95) | 42,745 | 252 | 9833.32 | 25.63 (22.65, 28.99) | 42,746 | 327 | 18712.01 | 17.48 (15.68, 19.48) | 42,750 | 480 | 35079.03 | 13.68 (12.51, 14.96) |
| *Stratifications by:* |  |  |  |  |  |  |  |  |  |  |  |  |  |  |  |  |  |  |  |  |
| **Age (years)** |  |  |  |  |  |  |  |  |  |  |  |  |  |  |  |  |  |  |  |  |
| ≥18 - 55 | 15,181 | 17 | 179.62 | 94.64 (58.83, 152.24) | 15,181 | 25 | 1251.95 | 19.97 (13.49, 29.55) | 15,181 | 36 | 3498.67 | 10.29 (7.42, 14.26) | 15,181 | 44 | 6673.80 | 6.59 (4.91, 8.86) | 15,181 | 61 | 12537.16 | 4.87 (3.79, 6.25) |
| 56 - 65 | 11,588 | 22 | 146.04 | 150.64 (99.19, 228.78) | 11,590 | 39 | 956.04 | 40.79 (29.80, 55.83) | 11,591 | 53 | 2662.86 | 19.90 (15.21, 26.05) | 11,591 | 70 | 5058.52 | 13.84 (10.95, 17.49) | 11,593 | 105 | 9446.15 | 11.12 (9.18, 13.46) |
| 66 - 75 | 11,438 | 51 | 157.62 | 323.56 (245.90, 425.74) | 11,438 | 71 | 942.84 | 75.30 (59.68, 95.02) | 11,438 | 90 | 2634.55 | 34.16 (27.79, 42.00) | 11,438 | 127 | 5009.82 | 25.35 (21.30, 30.17) | 11,440 | 190 | 9383.93 | 20.25 (17.56, 23.34) |
| 76 - <86 | 4,535 | 36 | 66.74 | 539.42 (389.10, 747.82) | 4,535 | 51 | 372.57 | 136.89 (104.03, 180.12) | 4,535 | 73 | 1037.24 | 70.38 (55.95, 88.53) | 4,536 | 86 | 1969.88 | 43.66 (35.34, 53.93) | 4,536 | 124 | 3711.79 | 33.41 (28.02, 39.84) |
| **Sex** |  |  |  |  |  |  |  |  |  |  |  |  |  |  |  |  |  |  |  |  |
| Male | 20,404 | 64 | 256.25 | 249.76 (195.49, 319.10) | 20,405 | 96 | 1680.10 | 57.14 (46.78, 69.79) | 20,405 | 129 | 4681.68 | 27.55 (23.19, 32.74) | 20,406 | 175 | 8898.68 | 19.67 (16.96, 22.81) | 20,409 | 258 | 16640.24 | 15.50 (13.72, 17.52) |
| Female | 22,325 | 62 | 293.61 | 211.16 (164.63, 270.84) | 22,326 | 90 | 1842.21 | 48.85 (39.74, 60.07) | 22,327 | 123 | 5148.73 | 23.89 (20.02, 28.51) | 22,327 | 152 | 9807.71 | 15.50 (13.22, 18.17) | 22,328 | 222 | 18427.89 | 12.05 (10.56, 13.74) |
| Unknown | 13 | 0 | 0.17 | 0.00 (0.00, 0.00) | 13 | 0 | 1.10 | 0.00 (0.00, 0.00) | 13 | 0 | 2.91 | 0.00 (0.00, 0.00) | 13 | 0 | 5.62 | 0.00 (0.00, 0.00) | 13 | 0 | 10.90 | 0.00 (0.00, 0.00) |
| **Race** |  |  |  |  |  |  |  |  |  |  |  |  |  |  |  |  |  |  |  |  |
| White | 38,246 | 113 | 487.13 | 231.97 (192.91, 278.94) | 38,248 | 160 | 3154.63 | 50.72 (43.44, 59.22) | 38,249 | 218 | 8814.21 | 24.73 (21.66, 28.24) | 38,250 | 286 | 16787.98 | 17.04 (15.17, 19.13) | 38,254 | 419 | 31522.14 | 13.29 (12.08, 14.63) |
| Black or African American | 2,545 | 9 | 36.89 | 243.99 (126.95, 468.92) | 2,545 | 17 | 210.81 | 80.64 (50.13, 129.72) | 2,545 | 23 | 585.70 | 39.27 (26.10, 59.09) | 2,545 | 28 | 1108.84 | 25.25 (17.44, 36.57) | 2,545 | 42 | 2063.04 | 20.36 (15.05, 27.55) |
| Asian | 212 | 0 | 3.33 | 0.00 (0.00, 0.00) | 212 | 0 | 17.43 | 0.00 (0.00, 0.00) | 212 | 1 | 49.07 | 20.38 (2.87, 144.69) | 212 | 2 | 93.98 | 21.28 (5.32, 85.09) | 212 | 3 | 172.13 | 17.43 (5.62, 54.04) |
| Other/Unknown | 1,739 | 4 | 22.68 | 176.39 (66.20, 469.96) | 1,739 | 9 | 140.54 | 64.04 (33.32, 123.08) | 1,739 | 10 | 384.34 | 26.02 (14.00, 48.36) | 1,739 | 11 | 721.21 | 15.25 (8.45, 27.54) | 1,739 | 16 | 1321.71 | 12.11 (7.42, 19.76) |
| **Type 1 Diabetes during baseline** |  |  |  |  |  |  |  |  |  |  |  |  |  |  |  |  |  |  |  |  |
| Yes | 270 | 1 | 3.85 | 259.59 (36.57, 1,842.88) | 270 | 2 | 22.56 | 88.64 (22.17, 354.43) | 271 | 3 | 64.50 | 46.51 (15.00, 144.22) | 271 | 4 | 123.85 | 32.30 (12.12, 86.06) | 271 | 9 | 231.14 | 38.94 (20.26, 74.83) |
| No | 42,472 | 125 | 546.17 | 228.86 (192.06, 272.72) | 42,474 | 184 | 3500.85 | 52.56 (45.49, 60.73) | 42,474 | 249 | 9768.82 | 25.49 (22.51, 28.86) | 42,475 | 323 | 18588.16 | 17.38 (15.58, 19.38) | 42,479 | 471 | 34847.88 | 13.52 (12.53, 14.79) |
| **Type 2 Diabetes during baseline** |  |  |  |  |  |  |  |  |  |  |  |  |  |  |  |  |  |  |  |  |
| Yes | 5,923 | 23 | 82.24 | 279.66 (185.84, 420.84) | 5,923 | 42 | 492.66 | 85.25 (63.00, 115.36) | 5,924 | 58 | 1394.58 | 41.59 (32.15, 53.80) | 5,924 | 81 | 2672.70 | 30.31 (24.38, 37.68) | 5,926 | 123 | 5026.02 | 24.47 (20.51, 29.20) |
| No | 36,819 | 103 | 467.78 | 220.19 (181.52, 267.09) | 36,821 | 144 | 3030.75 | 47.51 (40.35, 55.94) | 36,821 | 194 | 8438.74 | 22.99 (19.97, 26.46) | 36,822 | 246 | 16039.31 | 15.34 (13.54, 17.38) | 36,824 | 357 | 30053.01 | 11.88 (10.71, 13.18) |
| **Existing permanently implanted device or prosthesis during baseline** |  |  |  |  |  |  |  |  |  |  |  |  |  |  |  |  |  |  |  |  |
| Yes | 1,242 | 0 | 17.80 | 0.00 (0.00, 0.00) | 1,242 | 1 | 102.71 | 9.74 (1.37, 69.12) | 1,242 | 1 | 287.33 | 3.48 (0.49, 24.71) | 1,242 | 4 | 547.00 | 7.31 (2.74, 19.48) | 1,242 | 8 | 1019.47 | 7.85 (3.92, 15.69) |
| No | 41,500 | 126 | 532.23 | 236.74 (198.81, 281.91) | 41,502 | 185 | 3420.70 | 54.08 (46.82, 62.47) | 41,503 | 251 | 9545.98 | 26.29 (23.23, 29.76) | 41,504 | 323 | 18165.01 | 17.78 (15.94, 19.83) | 41,508 | 472 | 34059.56 | 13.86 (12.66, 15.17) |
| **Total length of hospital stay** |  |  |  |  |  |  |  |  |  |  |  |  |  |  |  |  |  |  |  |  |
| 1-5 days | 37,077 | 60 | 393.57 | 152.46 (118.38, 196.36) | 37,079 | 104 | 3053.66 | 34.07 (28.11, 41.28) | 37,080 | 148 | 8514.74 | 17.39 (14.80, 20.42) | 37,081 | 209 | 16198.14 | 12.91 (11.27, 14.78) | 37,084 | 336 | 30364.03 | 11.07 (9.95, 12.32) |
| 6-10 days | 4,530 | 43 | 98.86 | 434.95 (322.58, 586.48) | 4,530 | 57 | 375.63 | 151.74 (117.05, 196.72) | 4,530 | 70 | 1054.52 | 66.38 (52.52, 83.90) | 4,530 | 80 | 2012.51 | 39.75 (31.93, 49.49) | 4,530 | 99 | 3774.44 | 26.23 (21.54, 31.94) |
| > 10 days | 1,135 | 23 | 57.60 | 399.33 (265.37, 600.93) | 1,135 | 25 | 94.11 | 265.64 (179.49, 393.12) | 1,135 | 34 | 264.06 | 128.76 (92.00, 180.20) | 1,135 | 38 | 501.35 | 75.79 (55.15, 104.17) | 1,136 | 45 | 940.55 | 47.84 (35.72, 64.08) |
| **Use of implanted material during surgery on the same day as index surgery** |  |  |  |  |  |  |  |  |  |  |  |  |  |  |  |  |  |  |  |  |
| Yes | 3,095 | 11 | 44.45 | 247.47 (137.05, 446.87) | 3,095 | 16 | 255.45 | 62.63 (38.37, 102.24) | 3,095 | 19 | 714.26 | 26.60 (16.97, 41.70) | 3,095 | 32 | 1373.09 | 23.31 (16.48, 32.96) | 3,095 | 44 | 2626.26 | 16.75 (12.47, 22.51) |
| No | 39,647 | 115 | 505.58 | 227.46 (189.47, 273.08) | 39,649 | 170 | 3267.96 | 52.02 (44.76, 60.46) | 39,650 | 233 | 9119.06 | 25.55 (22.47, 29.05) | 39,651 | 295 | 17338.92 | 17.01 (15.18, 19.07) | 39,655 | 436 | 32452.77 | 13.43 (12.23, 14.76) |
| **Medical history of stroke** |  |  |  |  |  |  |  |  |  |  |  |  |  |  |  |  |  |  |  |  |
| Yes | 291 | 39 | 4.59 | 8,494.19 (6,206.12, 11,625.81) | 292 | 58 | 20.47 | 2,833.29 (2,190.40, 3,664.87) | 292 | 77 | 54.44 | 1,414.27 (1,131.18, 1,786.22) | 292 | 84 | 100.04 | 839.70 (678.03, 1,039.92) | 292 | 106 | 177.45 | 597.36 (493.81, 722.62) |
| No | 42,451 | 87 | 545.43 | 159.51 (129.28, 196.80) | 42,452 | 128 | 3502.94 | 36.54 (30.73, 43.45) | 42,453 | 175 | 9778.87 | 17.90 (15.43, 20.75) | 42,454 | 243 | 18611.98 | 13.06 (11.51, 14.81) | 42,458 | 374 | 34901.58 | 10.72 (9.68, 11.86) |
